# Mechanisms Regulating Stemness and Differentiation in Embryonal Carcinoma Cells

**DOI:** 10.1155/2017/3684178

**Published:** 2017-03-08

**Authors:** Gregory M. Kelly, Mohamed I. Gatie

**Affiliations:** ^1^Department of Biology, Molecular Genetics Unit, Western University, London, ON, Canada; ^2^Collaborative Program in Developmental Biology, Western University, London, ON, Canada; ^3^Department of Paediatrics and Department of Physiology and Pharmacology, Western University, London, ON, Canada; ^4^Child Health Research Institute, London, ON, Canada; ^5^Ontario Institute for Regenerative Medicine, Toronto, ON, Canada; ^6^The Hospital for Sick Children, Toronto, ON, Canada

## Abstract

Just over ten years have passed since the seminal Takahashi-Yamanaka paper, and while most attention nowadays is on induced, embryonic, and cancer stem cells, much of the pioneering work arose from studies with embryonal carcinoma cells (ECCs) derived from teratocarcinomas. This original work was broad in scope, but eventually led the way for us to focus on the components involved in the gene regulation of stemness and differentiation. As the name implies, ECCs are malignant in nature, yet maintain the ability to differentiate into the 3 germ layers and extraembryonic tissues, as well as behave normally when reintroduced into a healthy blastocyst. Retinoic acid signaling has been thoroughly interrogated in ECCs, especially in the F9 and P19 murine cell models, and while we have touched on this aspect, this review purposely highlights how some key transcription factors regulate pluripotency and cell stemness prior to this signaling. Another major focus is on the epigenetic regulation of ECCs and stem cells, and, towards that end, this review closes on what we see as a new frontier in combating aging and human disease, namely, how cellular metabolism shapes the epigenetic landscape and hence the pluripotency of all stem cells.

## 1. Introduction

We have just celebrated the 10th anniversary of the Takahashi-Yamanaka report on induced pluripotent stem cells, where introducing four transcription factors (Oct4, Sox2, Klf4, and c-Myc) was sufficient to reprogram fibroblasts towards pluripotent stem cells [1]. Although this work is a milestone in itself, paving the way for research into furthering our understanding of development and disease [2, 3], we must be reminded that most of the investigations into embryonic stem cells (ESCs) and cancer stem cells (CSCs) were preceded by those that focused on teratomas and teratocarcinomas [4–10]. The history is attention-grabbing, as over the last two thousand years teratomas have been attributed to everything from lucky omens, consorting with demons and the devil, participating in inappropriate sexual behavior, and incomplete twinning [5, 11]. Depending on the source, we know the word is derived from the Greek* terato(s)* [12],* teras* [13], or* teraton* [14] meaning monster and* oma* from onkoma or swelling [15] and was first reported in the mid-1860s by Rudolf Virchow [16]. Teratomas, which are benign germ cell tumors that contain cells derived from one or more of the three germ layers, develop spontaneously in the testes of the 129 family of inbred mouse strains, or they can be induced in adult mice when the genital ridges of embryos or early embryos themselves are ectopically transplanted into the testes or kidney [17, 18]. How teratomas develop has been the topic of much debate and is well beyond the scope of this review. However, we would be remiss if we did not note the recent findings that Cyclin D1, a target of canonical Wnt/*β*-catenin signaling, plays a key role in predisposing germ cells to switch their developmental potential to form teratomas containing somatic tissues [19]. These teratomas represent “an intersection of pluripotency, differentiation and cancer biology” [20]. Teratocarcinomas contain early embryo-like cells called embryonal carcinoma cells (ECCs) that share three distinct features: (1) they are malignant; (2) they can differentiate into any of the three germ layers or extraembryonic tissue; and (3) they can develop normally when injected into the blastocyst [21, 22]. Although ECCs cells can be propagated following transfer of individual cells [23], the ability to culture them in vitro and their loss of “multipotentiality” [24] set the stage for the studies that followed. Pioneering work by Ralph Brinster, Richard Gardner, Michael McBurney, Beatrice Mintz, Virginia Papaioannou, and many others recognized the importance of ECCs, and their ability as noted by François Jacob, to adopt a normal fate when injected into host mouse blastocysts [25, 26]. In those early days, many were not fully aware that the attributes of these in vitro model systems would be so instrumental in contributing to studies that delved into trying to understand how ESCs and CSCs remain in a pluripotent state and how intrinsic and extrinsic factors reverse the ability of these cells to self-renew to allow them to differentiate into new lineages. In fact, the suggestion that the genetics of ECCs would uncover genes involved in stem cell self-renewal and pluripotency [27] only serves to underscore the importance of ECC lines. These lines have been and continue to be studied extensively [28–31], and although similarities and differences exist between them, as well as between ECCs and those representative of ESCs and CSCs, this review will focus almost exclusively on two ECC lines from mouse (F9 and P19) and one from human (NTERA-2) and how various pathways influence their pluripotency state. In light of the considerable number of studies generated using these lines, especially in regard to differentiation, which warrants its own review and has been presented in part for P19 cells [32], we have purposely concentrated our efforts to highlight what has been learned about self-renewal and pluripotency from ECCs, and in some cases how these studies have extended to ESCs and CSCs.

## 2. Embryonal Carcinoma Cells

The utility of ECCs as a proxy for the study of early mammalian development and neoplasia was recognized long before we began asking questions regarding pluripotency and self-renewal [4, 33, 34]. Not only were these early studies instrumental in uncovering many of the in vivo mechanisms that govern development [35], but also they led to the widely accepted theory on the process of cancer development [31].

### 2.1. F9 Teratocarcinoma Cells

F9 teratocarcinoma cells, one widely used mouse ECC line developed from another teratocarcinoma [36], give rise to tumors consisting almost exclusively of undifferentiated cells [21, 37, 38]. F9 cells exhibit a pseudodiploid karyotype composed of 38 acrocentric and 1 metacentric chromosomes, and a G1 and S phase of approximately 8 hours [37]. Once considered nullipotent, as they have lost the ability to differentiate spontaneously [37], studies would later reveal that F9 cells are capable of differentiating into extraembryonic endoderm-like cells [39, 40], and evidence would indicate that they share many characteristics of ESCs [41]. Subsequent studies reported that F9 cells can be induced by all-*trans* retinoic acid (RA) [42], a natural derivative of vitamin A (retinol), thus setting the stage for a plethora of studies to follow [43].

### 2.2. P19 Cells

P19 cells, another mouse ECC line, were derived from a 7.5-day* post coitum* embryo that was transplanted into the testis of an adult mouse [44–46]. These cells, which represent a population at a later stage of development than the F9 cells [38], are pluripotent and resemble epiblast stem cells. P19 cells have a male euploid karyotype (40 and XY), and much like F9 cells are considered nullipotent [47]. P19 cells can differentiate into neurons, glial cells, and fibroblasts when treated with RA or into skeletal and cardiac muscle when treated with DMSO [32, 48–52]. While many studies have shed light on the similarities between F9 and P19 cells, details would eventually emerge to indicate that differences in gene regulation allow them to break from pluripotency and differentiate [53–55]. For instance, F9 cells have greater reprogramming capabilities than P19 cells, and this is probably due to differences in the levels of the master pluripotency gene* Sox2*.

### 2.3. NTERA-2 Cells

While most studies with ECC lines have focused on those of mouse origin, the NTERA-2 cell line is a human ECC line first established in the 1980s from a testicular teratocarcinoma from a 22-year-old Caucasian male [56]. NTERA-2 cells exhibit a hypotriploid karyotype with a modal chromosome number of 63 [57]. NTERA-2 cells, like mouse ECCs, respond to RA and differentiate towards a neural lineage [5, 58–67]. Furthermore, NTERA-2 cells differentiate into nonneural epithelial cells when treated with bone morphogenic protein-2 (BMP-2), whereas 27X-1 cells, another human ECC line, differentiate into extraembryonic endoderm when exposed to BMP-2 or RA [68, 69]. It is obvious that differences within ESCs [70] and ECC lines exist (Table 1); however, one unifying concept is in their ability to respond to RA, which leads to a loss of pluripotency factors resulting in differentiation towards certain lineages.

## 3. Retinoic Acid: Lessons from the Inducer

RA is a potent teratogen and an important endogenous regulator of proper and extraembryonic endoderm [71]. RA signaling has many diverse roles in the differentiation of ECCs [72, 73], most often leading to extraembryonic endoderm-like lineages, which in itself has led to the derivation of the extraembryonic-like ECC lines PYS2 and END2 cell [74]. RA-induced differentiation is accompanied by changes in gene expression in F9 cells [75–80], P19 cells [80, 81], and NTERA-2 cells [82]. Genes on this exhaustive list include* c-Myc* [83, 84] and* Int-1* (later renamed* Wnt1*), which have become the focus of many subsequent cancer-related studies. In the case of* c-Myc* in P19 cells, its expression following RA treatment follows two transient increases at 3 h and 48 h, and then it drops below basal levels by 144 h [83]. In contrast,* c-Myc* expression in F9 cells declines with RA-induced differentiation [78] comparable to ESCs [85]. We now know c-*Myc* is downstream of the Wnt targetome [86], but it was the discovery of* Wnt1* itself that was exciting to many in the scientific community as it linked* Drosophila* embryogenesis and the Wingless protein to protooncogenes and cancer [87–89]. Later reports have highlighted* Wnt1 *and other* Wnt* genes expression during RA-induced differentiation in P19 cells [90–92], F9 cells [78, 93, 94], and NTERA-2 cells [95, 96]. These early discoveries led to the assembly of complex cell signaling pathways and gene networks linked to ECC differentiation, and these were to be the platform that many have since used to identify the crosstalk and autoregulatory loops that exist within and between ECCs and ESCs [97–103]. It is interesting that while many of these studies revealed that RA must repress certain genes during differentiation, little discussion at the time linked these genes to self-renewal and stemness in ECCs. In fact, despite the irony that stemness genes including* c-Myc* [83, 104–107],* Oct3/4* [108–110], and* Sox2 *[111–113] had already been identified in ECCs, their specific roles in self-renewal and pluripotency would not be elucidated until later [27, 114]. Meanwhile, studies showing expression of genes such as* K-fgf* and* Hst-1* [115–117],* TGFα*, and* LAMIN A/C* [117–121] provided the framework that gene activity was sufficient and necessary to keep ECCs in the pluripotent state. Two genes linked to stemness and pluripotency are* Rex1* (*Zfp42*), encoding a zinc finger transcription factor, and* Ccnd1*, encoding Cyclin D1.* Rex1* expression is not detected in undifferentiated P19 cells [122–124]; however, it is induced when* Nanog* is overexpressed [122, 125]. Similarly, Nanog controls the expression of* Ccnd1*, as seen when* Nanog* is depleted or overexpressed in P19 cells [125] and by retinoids in NTERA-2 cells, which promotes the ubiquitination and degradation of Cyclin D1 [67]. In addition to these studies and others involving* c-Myc* [104, 126–130],* Oct4* [110, 131–133], and* Sox2* [111, 112], further evidence that pluripotency genes must be developmentally regulated in the early embryo came from reports that* Nanog* [134, 135] and* Foxm1* [136] are downregulated in P19 and F9 cells in response to RA treatment. Foxm1, a member of the Forkhead box of transcription factors, is an interesting example as we now know it plays pivotal roles in cell proliferation, differentiation, and self-renewal and acts downstream of canonical Wnt/*β*-catenin signaling [137, 138]. This downregulation of* Oct4*,* Nanog*, and* Sox2* is also seen in NTERA-2 cells treated with RA [139], which together with reports on the effects of deregulating* c-Myc *expression [140] and* ZNF536,* encoding a novel zinc finger protein [141] in F9 and P19 cells, respectively, underscores the importance of activating, regulating, and maintaining self-renewal and pluripotency genes in the undifferentiated state. Thus, while much of the focus was initially on ways to differentiate ECCs, subsequent efforts were underway to identify pluripotency factors that would attenuate differentiation, thereby maintaining stemness.

## 4. Pluripotency Factors and Signaling Crosstalk

### 4.1. Leukemia Inhibiting Factor (LIF)

**Figure 1:**
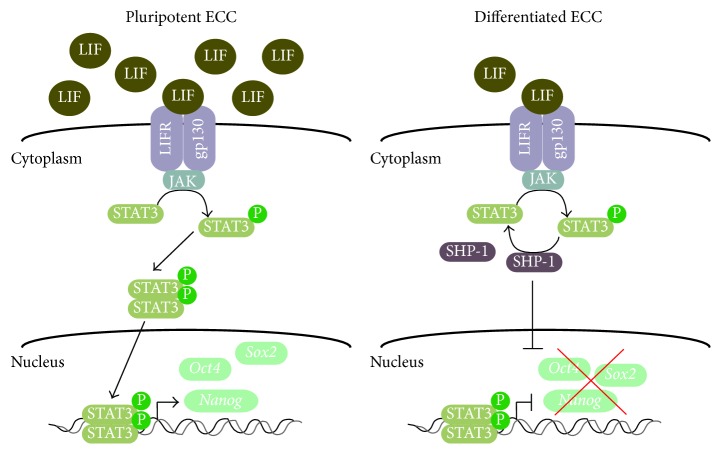
LIF signaling maintains ECCs pluripotency. In pluripotent ECC lines, LIF binds to the LIF receptor (LIFR) and gp130 recruiting Janus Kinase (JAK), which in turn phosphorylates and activates Signal Transducer and Activator of Transcription 3 (STAT3). Phosphorylated STAT3 homodimerizes and translocates to the nucleus promoting the expression of pluripotency genes encoding OCT4, SOX2, and NANOG. During differentiation, LIF levels decline substantially leading to decreased phosphorylated STAT3, which is augmented by the tyrosine-specific protein phosphatase SHP-1. The decline in active STAT3 reduces the expression of pluripotency genes.

Differentiating inhibiting activity (DIA)/leukemia inhibiting factor (LIF) produced by feeder cells or medium conditioned by Buffalo rat liver cells can block differentiation and promote stemness [124, 142–144]. Interestingly, ECCs harbor components responsible for LIF signaling [145, 146] and can secrete factors that support self-renewal of ESCs, but, unlike ESCs, they can maintain pluripotency in the absence of LIF or feeder layers [124, 147]. LIF belongs to the IL-6 family of cytokines, which contain IL-11, oncostatin M, ciliary neurotrophic factor, and others [148], all of which can signal through the JAK/STAT3 (Janus Kinase/Signal Transducer and Activator of Transcription) pathway. LIF activation in P19 cells [149, 150] blocks endodermal and mesodermal differentiation [114] and potentiates RA-induced neural differentiation [144, 151]. LIF has no apparent effect on NTERA-2 cells [114], and although there was early debate as to whether or not it had an effect on F9 cells [152, 153], recent evidence indicates that it blocks the ability of F9 cells to differentiate towards an extraembryonic lineage when induced by RA [154]. Moreover, STAT3 in F9 cells is regulated by Src-homology protein tyrosine phosphatase-1 (SHP-1), leading to its dephosphorylation and subsequent decrease in* Nanog* expression, which, as noted by the authors, restricts the expansion of the epiblast at implantation (Figure 1) [155].

### 4.2. Nanog

The homeodomain transcription factor Nanog plays an essential role in maintaining stem cell pluripotency and self-renewal. The* Nanog* promoter is well characterized, and although it is known to contain Oct4 and Sox2 binding sites, the early studies were contradictory with the report that Oct4 acts alone to induce* Nanog* expression [156], while another noted that both Oct4 and Sox2 were required [157].* Nanog* overexpression in F9 cells maintains them in the undifferentiated state, as evident by the upregulation of* Oct4* and* SSEA-1* and downregulation of markers of differentiation including* Gata-6*,* Gata-4*,* Hnf1β*, and* LamininB1 *[135]. The* Nanog* promoter contains one negative and two positive* cis*-regulatory elements that are active in F9 and ES cells, but only one positive element is active in P19 cells [134]. This example not only underscores the complexity of Nanog regulation, but also highlights the differences that exist between ECC lines. Negative feedback loops that regulate ESC pluripotency involving Oct4, FoxD3, and Nanog are known [158]. Furthermore, the presence of one of these loops whereby the* Nanog* promoter is negatively regulated by its own ectopic expression in ECCs would indicate that the pathway is even more complex than first thought [159]. Similarities in the pathway controlling Nanog expression exist between F9 and P19 cells, but because their Nanog levels differ, as well as Sox2, so too does their pluripotency state [41, 134]. Nevertheless, Nanog in ECCs is regulated by a Sox2 : Oct4 ratio [159] as well as the interplay between* Oct4* and* Rex1*, which are both involved in the maintenance of self-renewal downstream of Nanog [125].

### 4.3. Oct4

The crosstalk and feedback within proteins encoded by self-renewal and pluripotency genes are highlighted by the regulation of Oct4, considered as the master regulator of totipotency [160]. Oct4 has many roles in gene regulation, and its own positive and negative regulation is the topic of many studies. For instance, Nspc1, a polycomb protein and a transcriptional repressor that is highly expressed in undifferentiated P19 cells, directly activates the* Oct4* promoter [161], which in itself gets negatively regulated following RA treatment [78, 108, 162–165]. Once* Oct4* is transcribed and translated in F9 cells,* Rex1* expression is upregulated [166, 167], and similarly, in P19 cells, the* Rex1* promoter is activated by either Oct4 or Sox2 when Nanog is overexpressed [122]. The* Rex1* promoter is also activated in differentiated P19 cells when Oct4 is overexpressed [166]. However, while it may seem contradictory, high levels of Oct4 downregulate* Rex1 *expression in F9 cells [166], which again highlights the importance of the cellular environment and context.

### 4.4. Wnt Signaling and miRNAs

**Figure 2:**
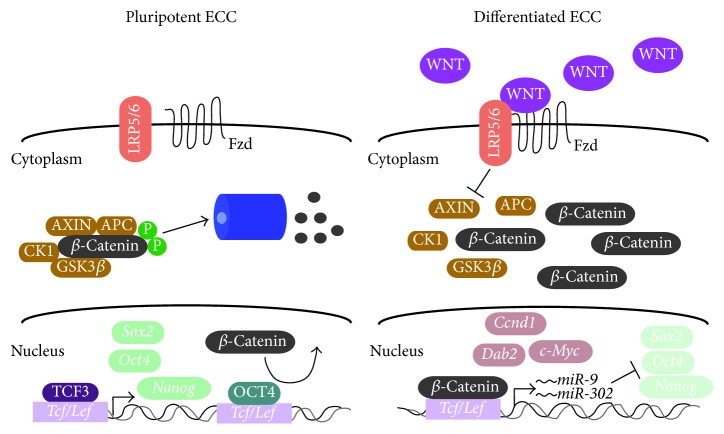
The dual role of WNT/*β*-catenin signaling ECCs stemness and differentiation. In the absence of WNT ligand, *β*-catenin is phosphorylated and degraded by the proteasome; subsequently, TCF3, a transcriptional repressor of WNT target genes, translocates to the nucleus and upregulates pluripotency genes. TCF3-dependent upregulation of OCT4 allosterically binds to TCF/LEF, preventing *β*-catenin from binding, which attenuates WNT signaling and differentiation. In the presence of WNT, AXIN is downregulated leading to the dissociation of the destruction complex, which results in the accumulation of *β*-catenin in the cytoplasm and subsequent translocation to the nucleus where it binds to TCF/LEF proteins. As a result, WNT target genes including* Dab2*,* Ccnd1*, and* c-Myc* are upregulated leading to cell differentiation. *β*-catenin-TCF/LEF interactions also result in the increase in* miR-9 *and* miR-302* expression, which in turn downregulate the expression of pluripotency genes.

Although studies indicate that Wnt signaling suppresses pluripotency and promotes differentiation,* Oct4* overexpression in P19 cells suppresses canonical Wnt signaling [168]. Nevertheless, canonical Wnt signaling is linked to Oct4 and pluripotency as evident by the fact that the downregulation of* Oct4* expression occurs when T-cell factor 3, Tcf3, serving as a transcriptional repressor in the absence of *β*-catenin, is overexpressed in F9 cells (Figure 2) [169]. This supports what was described earlier where Wnt is induced and Axin, a negative regulator of canonical Wnt signaling, declines in RA-treated F9 and P19 cells [88, 91–93, 170]. Another level impacting pluripotency and stemness involves microRNAs (miRNAs), which together with Oct4, Sox2, and Nanog are regulated positively by Wnt signaling (Figure 2) [171]. In one case this regulation involves the* mir-302* gene, which encodes a cluster of 5 microRNAs (miRNAs) that are highly expressed in undifferentiated NTERA-2 cells and P19 cells [172, 173]. Oct4 can bind directly to* miR-302* and upregulate its expression [171], while canonical Wnt signaling regulates* mir-302 *expression involving 3 TCF/LEF binding sites. In the latter, knocking down *β*-catenin leads to decreased expression of* mir-302*, whereas knocking down Tcf3 produces the opposite effect [174], which promotes the expression of pluripotency genes in F9 and P19 cells [154, 175]. Other miRNAs play a role in regulating stemness and differentiation of mouse and human ECCs (Table 2), including* miR-9, *whose expression not only increases with differentiation, but also serves to repress* Sox2* in NTERA-2 cells [176]. Other examples include* miR-124, *where elevated levels in P19 cells promote neuronal differentiation by suppressing* Ezh2*, a histone methyltransferase, which represses genes involved in neurogenesis [177]. While many miRNAs are known to impact the ability of ECCs to remain pluripotent [172, 177–182], other modes of regulation, including that by PI3K/AKT signaling, can influence pluripotency and stemness.

### 4.5. PI3K/AKT Signaling

The PI3K/AKT signaling pathway is well known to have a key role in cell growth, proliferation, metabolism, and survival [183]. This list can be added to as RA and the negative regulation of the* Oct4* promoter are accompanied by an increase in AKT signaling in F9 cells [184] and the suppression of PTEN in P19 cells [185]. Furthermore, the phosphorylation of Oct4 and Klf4 by AKT leads to their degradation by the ubiquitin-proteosome system, which promotes the loss of pluripotency in F9 cells [186]. Although RA was previously described as an inducer of differentiation, its presence promotes AKT-dependent phosphorylation of the chromatin remodeler SATB1, which binds to SOX2 thereby preventing it from associating with Oct4 to maintain pluripotency. In addition, and contrary to what was described earlier [186], active AKT signaling in P19 cells induces a transient increase in* Nanog* expression [187], which seems counterintuitive because AKT inhibits pluripotency markers. However, it is more complex than this as Oct4 in ECCs can bind to the human* AKT1* promoter, and this is dependent on its phosphorylation state controlled by AKT itself [188]. In fact, these authors found that the stabilization of Oct4 through AKT phosphorylation not only promotes its dissociation from the* AKT1* promoter, but also facilitates its interaction with Sox2 to upregulate* Nanog* expression. Thus, PI3K/AKT signaling is another means by which pluripotency is dictated in ECCs. However, other mechanisms downstream of signaling pathways such as epigenetic modifications also play a role in regulating stemness and differentiation [189].

## 5. Epigenetic Modifications

Understanding how global changes in gene expression are required to maintain ECCs in a pluripotent state has been a daunting task, one that is nearly eclipsed when considering the roles epigenetic modifiers and chromatin remodelers have on regulating these genes. The complexity imposed by these control mechanisms, especially as they relate to ESC pluripotency and differentiation, is evident by the many recent reviews and their historical account of the field [190–200]. While many epigenetic modifications exist, we will focus on the most common: DNA methylation and histone modifications by acetylation, which intricately link pluripotency genes and microRNAs in germ cell tumor development [201–208] to cancers and other diseases [209–215]. The molecular details of how these modifications occur and the effects imparted by these changes are presented elsewhere [193, 216–219].

### 5.1. DNA Methylation

**Figure 3:**
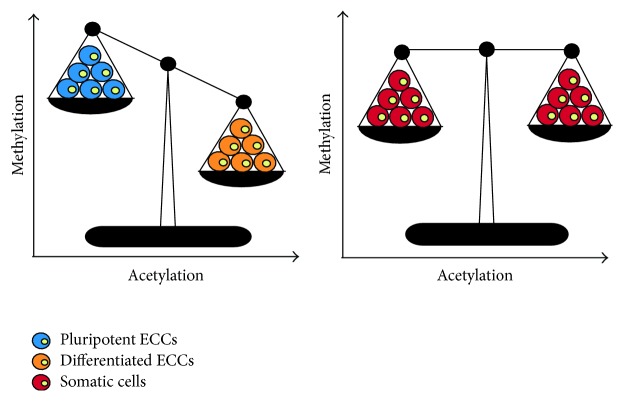
Global methylation and acetylation pattern of ECC lines during stemness and differentiation. Pluripotent ECCs are methylated and deacetylated yielding a low expression profile of differentiation markers. The dimerization of Jun Dimerization Protein 2 (JDP2) results in the recruitment of Histone Deacetylase 3 (HDAC3), which interacts and inhibits Histone Acetylases (HATs) from activating differentiation genes. Pluripotency is lost when DNA methyltransferase 1* (Dnmt1)* expression and activity is reduced, resulting in the demethylation of differentiation-inducing genes including* Vimentin*,* Laminin B1, Collagen IV,* and* Endo A*. Similarly, HDAC activity is reduced as the p300 complex displaces the JDP2/HDAC3 complex, resulting in the recruitment of HATs and initiation of c-Jun transcription leading to differentiation.

Methylation of the fifth cytosine of CpG islands on the promoter of various genes is conserved in Eukaryotes [220]. Global methylation patterns in somatic cells are relatively stable and well characterized (Figure 3); however, DNA methylation or demethylation is tightly regulated and highly dynamic during embryo development [221, 222]. While multiple studies have highlighted the importance of DNA methylation in the maintenance of stemness and differentiation in ESCs [223–233], some of the first reports were with ECCs.

#### 5.1.1. Methylation, Stemness, and Differentiation

These studies used 5-Azacytidine, which can induce differentiation of various ECCs by inhibiting DNA methyltransferases (DNMTs), though surprisingly not in F9 cells [234–237]. We have seen that F9 cells treated with a DNMT inhibitor do not differentiate and undergo apoptosis instead (unpublished data), which corroborates an earlier report [238]. Therefore, global demethylation in F9 cells is not sufficient to induce differentiation, even though it results in a demethylation profile like that seen following RA treatment [236, 239–241]; however, gene-specific demethylation pattern might vary. The methylation status of any gene can be misinterpreted, as in the case of those involved in the maintenance of pluripotency in F9 cells, which are hypomethylated and therefore transcriptionally active, while those induced with RA, including* Vimentin* [242],* Laminin B1* [243],* Collagen IV* [244], and* Endo B* [245], are hypermethylated making them inactive. Furthermore, the promoter of the* Thrombomodulin*, a marker of differentiation, gene shows a similar methylation pattern regardless of the differentiation status of the F9 cells. Thus, other mechanisms such as chromatin remodeling may also play a role in gene expression [246]. We know P19 cells treated with RA differentiate towards a neuronal lineage [49, 51], and this is marked by global demethylation [247], which is similar to what is seen in F9 cells treated with RA [239]. A reduction in DNMT protein levels and activity leads to reduced global and gene-specific methylation involved in differentiation seen in other ECCs [237, 239, 248, 249]. Specifically in P19 cells, the AP-1 regulatory site in the* Dnmt1* promoter is heavily methylated in the undifferentiated state [250]. When challenged by 5-Azacytidine, CpG islands upstream of the AP-1 site are demethylated leading to the recruitment of the Jun/Fos complex leading to expression of* Dnmt1*, which in turn methylates those same sites leading to transcriptional repression [250]. In fact, the methylation of regulatory regions in the* Dnmt1* promoter acts in a feedback mechanism, as sensors for the methylation capacity of the cell [250]. While downstream changes to methylation profiles have been linked extensively to pluripotency potential, much less attention has been given to upstream regulators. Early work has emphasized the importance of RAS and its downstream effectors [54, 251] on the role in global and site-specific demethylation by inducing the phosphorylation and activation of c-JUN, which binds to the* Dnmt1* promoter, inducing its expression and leading to differentiation [252]. These examples of consistent methylation trends, differences in DNMT activity, and activation of signaling pathways reveal the complexity underpinning the maintenance of stemness and differentiation in ECCs. Although much has been learned regarding the influence of DNA methylation on genes involved in differentiation, we know that similar mechanisms are in place for self-renewal. For instance, Oct4 and Nanog promote pluripotency in ESCs and ECCs, but it is important to note that the expression of these genes is regulated by methylation. The* Nanog* promoter in undifferentiated NTERA-2 cells is methylated 200 bp upstream of Oct4/Sox2 binding domains [253]. Similarly, the methylation status of the* c-Myc* promoter in F9 cells dictates the levels of the protein, which are high in the undifferentiated state but fall precipitously due to RA treatment [130]. This is recapitulated in human ESCs where high c-Myc levels maintain pluripotency in the absence of LIF/STAT3 [254]. Undifferentiated P19 cells maintain high levels of* Oct4* expression by promoting a low methylation profile on the* Oct4* locus [255, 256]. Taken together, these studies would indicate that the ratio of demethylation-to-methylation dictates the expression of pluripotency genes in ECCs, and thus the maintenance of stemness [255, 256]. If so, this role of methylation status could account for the heterogeneity in pluripotency as others and we have noted in ECCs populations.

### 5.2. RA Signaling and Methylation

While overwhelming evidence was presented earlier that RA induces ECCs differentiation, it is necessary to devote a brief description on how RA and retinoid synthesis and transport to their RAR/RXR are linked to the methylation status of promoters. During differentiation, the cellular RA binding protein 1 (CRABP-1) binds to RA and delivers it to RAR/RXR sites on the DNA of target genes [257]. RA induces CRABP-1 in P19 and F9 cells, yet the methylation pattern of its promoter remains unchanged, even in the presence of 5-Azacytidine, which does not induce* CRABP-1* expression [258]. Conversely, a methylated Histone H2B variant (TH2B) transfected into F9 cells gets demethylated, signifying that there is active histone gene expression in the undifferentiated state [259]. The* Th2b* gene, like many other housekeeping genes, maintains a low methylation profile, which is largely due to the protection of CpG islands imparted by the SP1 transcription factor [260, 261]. Whereas DNA methylation can play a role in transcriptional repression, the activation of the* H-2K* gene during differentiation is associated with hypermethylation, and this is evident from studies with 5-Azacytidine, which attenuates* H-2K* expression in F9 cells [262]. Since DNA methylation is not easily reversed, gene regulation may be better controlled temporally by the more labile and reversible modifications made to histones.

### 5.3. Chromatin Remodeling

Eukaryotic DNA with all its modifications is folded into nucleosomes, which are made up of histone octamers. Since resolving the nucleosome structure [263, 264], many histone modifications have been discovered [265–268]. These discoveries have provided us with a great understanding of how changes to chromatin availability directly or indirectly regulate gene expression. Although histone modifications by methylation are known to play a role in chromatin availability [269], we will focus on acetylation modifications in ECCs. In one example, the modification involves histone acetylation of lysine residues via histone acetyl-transferases (HATs), which weaken DNA-histone interactions allowing several proteins to dock and initiate transcription.

#### 5.3.1. Histone Modifications, Stemness, and Differentiation

P19 cells maintained in an undifferentiated state have high levels of nonacetylated histones (Figure 3), but this declines with RA induction [270, 271]. A similar situation occurs in NTERA-2 cells, where regulatory regions of* Oct4* and* Nanog* in differentiated cells are hypoacetylated, leading to the closed chromatin conformation and reduced expression [272]. The expressions of* Oct4* and* Nanog *are influenced by the knockdown of the* Brahma related gene*,* Brg1*, encoding a protein present in Brg-containing Switch/Sucrose NonFermentable (SWI/SNF) complexes [273]. In P19 cells, the involvement of Brg1 with the promyelocytic leukemia protein maintains an open chromatin conformation of the* Oct4* gene [274], whereas RA induces silencing and chromatin remodeling by receptor-interacting protein 140 [72, 274]. This silencing and remodeling displaces Brg1 for the Brahma- (Brm-) containing SWI/SNF complex on both the* Oct4* and* Nanog* promoters, thus silencing transcription [275]. Although comparable conditions exist in ESCs [276] this is not a universal phenomenon as histone acetylation near the* Nestin* locus accompanies RA-induced differentiation in P19 cells [277]. It is interesting to note that RAR^−/−^ F9 cells exhibit increased expression of* Slc38a* and* Stmn2*, which is normally associated with differentiated F9 cells due to hyperacetylation, suggesting that RA signaling might have a role in regulating histone modification [278]. Modifications by histone deacetylases (HDACs) are also intricately linked to RA signaling. HDACs antagonize HATs, as they remove acetyl groups from lysine residues, a modification that restores their positive charge and leads to chromatin stability that is largely associated with transcriptional repression. Jun Dimerization Protein 2 (JDP2) maintains stemness in F9 cells by serving as a transcription factor not only to recruit HDAC3, but also to have it interact and inhibit HATs [279]. During RA treatment, however, the p300 complex displaces JDP2/HDAC3 leading to acetylation, initiating c-Jun transcription and differentiation [280, 281]. Other examples of this regulation are seen with HDAC3 in P19 cells, which inhibits the autoactivation of the neuronal transcription factor NeuroD [270], or in studies using HDAC inhibitors, which show reduced* Nanog* expression in undifferentiated P19 and F9 cells, and* Esrrb*,* Klf2, *and* Rex1 *in ESCs [282]. Differentiated ECCs treated with an HDAC inhibitor and showing elevated pluripotency markers would suggest that HDAC activity could be largely regulated by the stemness state of a cell [282]. These reports and many others document the importance of HDACs in regulating chromatin availability, but the fact they also physically interact with pluripotency markers including Sox2 [282], Oct4 [283], and Nanog [284] reminds us of their involvement at other levels. In addition to the control conveyed directly by HDACs, chromatin remodeling of pluripotency genes is facilitated by other factors. For instance, in RA-induced differentiation of P19 cells,* Nanog* repression is the result of Foxa1, a member of the forkhead/winged-helix gene family induced by RA, to recruit the transcriptional corepressor Grg3, which belongs to the Gro/TLE/Grg family [285]. This potentiates subsequent recruitment of HDACs, together with Foxa1, to deacetylate histone 3 and repress the* Nanog* locus [285]. This type of multiplex control is not just reserved for* Nanog.* In differentiated P19 cells,* Oct4* expression initially increases, and in cooperation with histone H3 acetylation it induces* Meis1a* expression, which recruits HDAC1 directly to the* Oct4* promoter to subsequently reduce its activity [286]. Examples of this complex interplay between HDACs and other factors are well documented from ESC studies [198, 287, 288], and it would be of interest to investigate whether similar mechanisms are in place in ECCs.

### 5.4. RA Signaling and Histone Modification

Since HDAC deacetylation of key lysine residues on the regulatory regions of the RAR and RXR receptors would lead to their transcriptional repression, it is easy to envision given the network of genes regulated by RA how important this mechanism is to maintaining pluripotency. Butyrate inhibits histone deacetylation and promotes reversible morphological changes to F9 cells, but it does not induce differentiation [289]. Surprisingly, in the presence of cycloheximide, transcript and protein levels of differentiation markers were sustained in butyrate treated F9 cells [290]. Trichostatin A is another HDAC inhibitor that cannot induce P19 cell differentiation by itself but is able to when cells are cotreated with RA [291]. Like butyrate, treatment of Trichostatin A alone induces apoptosis in P19 cells, whereas cotreatment with RA induces RAR/RXR-induced transcription via histone acetylation [291]. In this study, the authors postulate that histone acetylation alone is not sufficient to induce differentiation, but it nevertheless primes ECCs for these events. Although the RAR/RXR response to acetylation plays a limited role during butyrate and Trichostatin A treatment, it more importantly suggests that other mechanisms are likely involved in the maintenance of stemness [292]. For example, CDK-associated Cullin 1 (CAC1), affects RA-induced differentiation by directly binding to RAR*α*, inhibiting its transcriptional activity in P19 cells [293, 294]. In this position, CAC1 recruits HDAC2, which deacetylates RAR2 to promote pluripotency [293]. While we have provided some examples of how epigenetic modifications influence gene regulation, we did not cover the source of cofactors required to induce such changes. The idea that epigenetic regulation is energetically demanding is often overlooked, and for that reason we will complete our discussion by addressing the fact that many of the cofactors involved are provided by cellular metabolism. Thus, the epigenome is intricately linked to the metabolome [295, 296], and any changes to one would be expected to have direct consequences on the other.

## 6. Cellular Metabolism

We begin this section with some of the earliest discussions to spotlight the contribution of the mitochondria and metabolism to pluripotency and stemness [296–299]. Mitochondria have many roles including its most well-known role, to produce ATP, and obligatory production of reactive oxygen species as the by-product of cellular metabolism. In addition, mitochondrial metabolism is linked to calcium signaling and apoptosis, all of which play a part in pluripotency in ESCs [300–303]. During oxidative phosphorylation (OXPHOS) one molecule of glucose generates 38 molecules of ATP, which in comparison to glycolysis generates 2 ATP molecules. Although glycolysis is inherently less efficient than OXPHOS, it does support and promote high cellular proliferation during embryonic development [304, 305], cancer initiation and progression [306, 307], and neurodegenerative disease states [308, 309]. A balance between glycolysis and OXPHOS metabolism has been considered as a “rheostat” for stem cell fate [310], as these processes that generate and utilize metabolites have an impact on changes in epigenetic modifications and cell signaling networks governing the equilibrium between pluripotency and differentiation [311–314]. The majority of ESCs tend to transition towards OXPHOS with differentiation [315, 316], although this is not universal [304] as evident in iPSCs derived from fibroblasts that revert to glycolysis [317]. These findings and others only serve to strengthen the argument that the metabolic state of a cell must be considered when discussing stemness and pluripotency, and we are reminded that some of the seminal studies that led to this account were first reported in ECCs in the 1990s.

**Figure 4:**
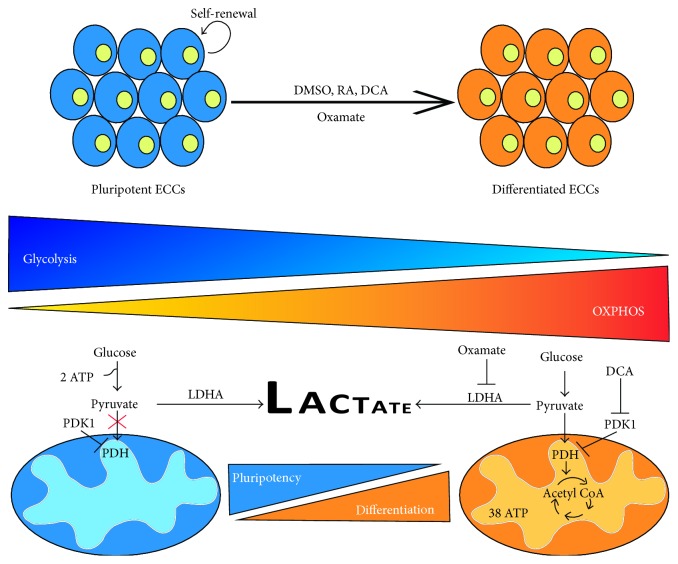
General overview of ECCs differentiation and the metabolic transition associated with the loss of stemness. ECCs can be differentiated towards neuronal and extraembryonic endoderm in the presence of retinoic acid, or cardiomyocytes in the presence of DMSO. With the differentiation process, others and we have observed a metabolic transition from glycolytic metabolism towards oxidative phosphorylation. Moreover, we can induce F9 cell differentiation towards an extraembryonic lineage using dichloroacetate (DCA), a pyruvate dehydrogenase kinase (PDK) inhibitor, or oxamate, a lactate dehydrogenase A (LDHA) inhibitor, which would indicate that the metabolic status of a cell determines whether it will remain pluripotent or if it will differentiate towards a specific lineage.

One such study examined Phosphofructokinase (PFK), a glycolytic enzyme that catalyzes the phosphorylation of fructose-6-phosphate to fructose 1,6-bisphosphate in the presence of ATP. Van Erp and colleagues (1990) reported that undifferentiated P19 cells show preference to* Pfk*-*L* expression, encoding an isoform that is more sensitive to activation by fructose 2,6-bisphosphate, and thus increasing the glycolytic rate [318]. Similar results were reported in highly proliferative cells for lactate dehydrogenase A (LDHA), which converts pyruvate to lactate [319] and pyruvate dehydrogenase kinase (PDK), which by phosphorylating and blocking the pyruvate dehydrogenase (PDH) complex inhibits the conversion of pyruvate to acetyl Co-A in the mitochondria [320]. Undifferentiated P19 cells have a strong glycolytic profile, which is correlated with high levels of Oct4, Sox2, and Nanog [320]. Similarly, we have found that F9 cells maintained in the undifferentiated state have high levels of LDHA and PDK1 (manuscript in preparation) (Figure 4). This parallels the increased lactate and pyruvate production seen in differentiating P19 cells [321]. The fate of pyruvate in these cells, however, differs significantly between the undifferentiated and differentiated cells, as when cells are grown in the presence of galactose, pyruvate is shuttled to the mitochondria and this is associated with reduced stemness [320]. It is interesting to note that undifferentiated P19 cells have fewer mitochondrial proteins [322] compared to their differentiated counterparts, even though both have similar mitochondrial DNA copy number [320]. This may not be a general phenomenon for all ECCs, as we have seen the opposite in F9 cells (manuscript in preparation). Similar mitochondrial DNA copy number between the undifferentiated and differentiated state is likely offset by mitochondrial activity, highly prevalent in differentiated ECCs, and substrate availability, which would fuel these mitochondria. What is perplexing is that P19 cells induced to form cardiomyocytes initially have low mitochondrial DNA content and ATP levels during the early stages of differentiation, but both eventually return to basal levels [323]. That these changes are not expected to occur during differentiation only reinforces the idea of the complex, nonuniversal nature and specificity of cellular metabolism in maintaining stemness or promoting differentiation. We have shown that F9 cells differentiate when OXPHOS metabolism is promoted (Figure 4), and this parallels what was reported earlier for P19 cells [320]. Likewise, while many have shown that the differentiation of ECCs is accompanied by a shift in the metabolic profile towards OXPHOS [320–322], it remains to be determined if this metabolic transition precedes the differentiation process or is the result of it. If we are to champion one or the other, it would probably be the former given the evidence that mouse embryonic fibroblasts reprogrammed using the Yamanaka factors transition to glycolytic metabolism prior to the induction of pluripotency [324]. This phenomenon, if general, would indicate that metabolism might be the master regulator of pluripotency and stemness and not just their slave in preventing differentiation.

## 7. Conclusion

Using ECCs as our platform we have highlighted some of the dramatic interplay that must exist between key genes and their regulators for these cells to remain pluripotent and to self-renew. Extrapolating many of these events to ESCs and CSCs in vivo has been fruitful in many cases, but as we have noted on several occasions, there are fundamental differences that preclude striking a unifying model. Other means of regulation are known to play key roles in ECC self-renewal and differentiation, including modifications imparted by reactive oxygen species (ROS) [325, 326], as well as the influence of ROS homeostasis in cellular programming [300, 327], cell cycle control as demonstrated in ESCs [328–333], as well as long noncoding RNAs in these cells [334–337]. In fact, signaling through Ca^2+^ channels in ECCs has also been reported to be involved in regulation [338]. Changes to the epigenome and chromatin remodeling, however, have garnered much attention. Together, we expect that in the future a better understanding of how these changes contribute to self-renewal and pluripotency in development will only serve to elucidate a major question on the minds of everyone—why do we get cancer?

## Figures and Tables

**Table 1:** Key features of ECC lines.

	F9 cells	P19 cells	NTERA-2 cells	References
Origin	Mouse	Mouse	Human	[37, 38, 57]
Colony morphology	Compact	Flat	Flat	[37, 38, 57]
Global methylation	Hypomethylated	Hypomethylated	Hypermethylated	[234, 248, 255]
Stemness genes	*Oct4, Sox2, Nanog, Klf4, Rex1, c-Myc*	*Oct4, Sox2, Nanog *	*Oct4, Sox2, Nanog, Dnmt3b, Fgf4, Rex1, Dppa5*	[58, 75–77, 81, 82]
LIF requirement	No	No	No	[122, 149]
RA-responsiveness	Yes	Yes	Yes	[37, 38, 59, 72]
Reprogramming efficiency	High	Low	Low	[41]
Teratoma formation	High	High	High	[22, 26]
Chimera contribution	High	High	No data available	[22, 26]
X chromosome status	X:0	Male	Male	[37, 38, 60]

**Table 2:** Trend of select miRNAs in P19 and NTERA-2 cells.

miRNA name	P19		NTERA-2		References
	P	D	P	D	
miR-9	−	+	−	+	[183]
miR-124	−	+	−	+	[178, 179, 183]
miR-125	−	+	=	=	[179]
miR-302	+	−	+	−	[179, 180, 183]
Let-7	−	+	−	+	[181]

P: pluripotent ECCs; D: differentiated ECCs

+: increase, −: decrease, and =: no change in levels.
